# Shaping Oncogenic Microenvironments: Contribution of Fibronectin

**DOI:** 10.3389/fcell.2024.1363004

**Published:** 2024-04-10

**Authors:** Gemma Guerrero-Barberà, Natalia Burday, Mercedes Costell

**Affiliations:** Departament of Biochemistry and Molecular Biology, Institut Universitari de Biotecnologia i Biomedicina, Universitat de València, Valencia, Spain

**Keywords:** fibronectin, cancer, extracellular matrix, mechanosignaling, alfa5 beta1 integrin, microenvironment

## Abstract

The extracellular matrix (ECM) is a complex network of proteins and glycans, dynamically remodeled and specifically tailored to the structure/function of each organ. The malignant transformation of cancer cells is determined by both cell intrinsic properties, such as mutations, and extrinsic variables, such as the mixture of surrounding cells in the tumor microenvironment and the biophysics of the ECM. During cancer progression, the ECM undergoes extensive remodeling, characterized by disruption of the basal lamina, vascular endothelial cell invasion, and development of fibrosis in and around the tumor cells resulting in increased tissue stiffness. This enhanced rigidity leads to aberrant mechanotransduction and further malignant transformation potentiating the de-differentiation, proliferation and invasion of tumor cells. Interestingly, this fibrotic microenvironment is primarily secreted and assembled by non-cancerous cells. Among them, the cancer-associated fibroblasts (CAFs) play a central role. CAFs massively produce fibronectin together with type I collagen. This review delves into the primary interactions and signaling pathways through which fibronectin can support tumorigenesis and metastasis, aiming to provide critical molecular insights for better therapy response prediction.

## Introduction

The glycoprotein fibronectin (FN) is particularly abundant in the microenvironment of malignant tumors (Castellani et al., 1986; Bae et al., 2013; Peng et al., 2022), is the first extracellular matrix (ECM) protein found in specific pre-metastatic niches (Medeiros et al., 2020), is present in the migration tracks used by metastatic cells (Erdogan et al., 2017) and its transcription is induced by hypoxia conditions in certain tumors (Mao et al., 2023). Consequently, FN is a constant presence during the process of matrix remodeling that occurs during solid tumor growth and metastatic foci formation. In these processes, the FN fibrillar structure harboring other ECM components, along with the expression of both FN splice isoforms and FN modifier and cross-linker enzymes, play critical roles in influencing angiogenesis, metastasis, and chemoresistance, thereby impacting the disease outcome. Expanding our comprehension of FN biology within tumors promises improved predictions of therapy responses. Furthermore, understanding these mechanisms has particular relevance for crafting experimental tumor models, such as patient-derived 3D-organoids cultivated *in vitro*.

In a number of tumors, as in 78% of the head and neck cancers, FN may be absent within the tumor mass but is abundant in the adjacent stroma (Beier et al., 2007). Within the stromal space, cancer-associated fibroblasts (CAFs) are major FN producers (Attieh et al., 2017). Originating mainly from resident tissue fibroblasts under tumor stimuli, CAFs express high levels of α-smooth muscle actin (αSMA) and exert contractile forces and focalized proteolysis, contributing to ECM remodeling and stiffening, and creating tracks that enable the invasion of cancer cells (Kalluri, 2016; Sahai et al., 2020). Therefore, FN is also a key factor mediating CAF functions (Jang and Beningo, 2019; Barbazan et al., 2023; Galbo et al., 2023), and its elevated levels in tumor ECM are often associated with poorer survival rates in cancer patients (Barkan and Chambers, 2011; Bae et al., 2013; Balanis et al., 2013; Fernandez-Garcia et al., 2014; Shinde et al., 2018).

**FIGURE 1:**
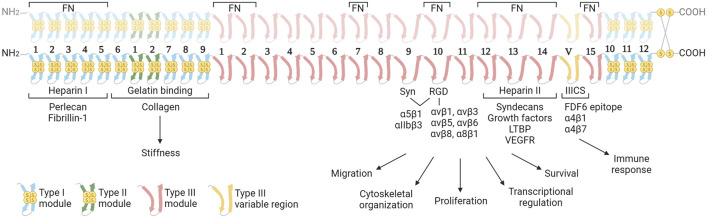
Structure of the fibronectin dimer with principal interacting molecules and potential biological function of the different modules in cancer. The FN consists of two almost identical subunits, each weighing between 230–270 kDa, and interconnected by two disulfide bonds. The dimer features three distinct types of modules (Types I, II and III), each characterized by unique elastic properties primarily influenced by the presence or absence of intramodule disulfide bonds. The image highlights the cell receptors and ECM molecules that interact with FN dimers. These interactions are determining the biological functions of FN in the context of cancer. In the upper part, the regions of interaction with other fibronectin molecules are indicated. Abbreviations: FN, fibronectin; Syn, synergy region; LTBP, latent transforming growth factor β binding protein; VEGFR, vascular endothelial growth factor receptor. Created with BioRender.com.

FN is a large, multidomain glycoprotein present in soluble form in blood (plasma FN; pFN) and as fibrillar networks in tissues (cellular FN; cFN). The pFN is synthetized by hepatocytes, and fibroblasts and endothelial cells are the major producers of cFN, but many other cell types can synthesize FN at lower levels. FN is encoded by a single gene (*Fn1*), is secreted as a dimer of two nearly identical subunits of 230–270 kDa, and linked by two disulphide bonds at the C-terminal region (Figure 1). FN monomers vary due to splicing, giving 20 different isoforms in humans and 12 in rodents (Goossens et al., 2009). FN structure includes three types of Ig-like repeats (FNI, FNII, and FNIII). FN includes 12 type I modules located at the N- and C-terminus of the protein. The FNI modules contain collagen (gelatin binding domain; GBD), fibrin and heparin I (HepI) binding sites. In FN there are two repeats of type II. Type I and II repeats contain two disulphide bonds and do not mediate cell interactions, but facilitate FN fibril formation. FN contains 15 type III repeats in addition to the alternatively spliced regions: the extra domains A (ED-A) and B (ED-B) and the type III connecting segment (IIICS). The FN type III secondary structure does not have disulphide bonds conferring high elasticity to the molecule (Potts and Campbell, 1996). FNIII modules contain several motifs for cell binding including ED-A, III9-10, and IIICS, which bind integrins, and the III12-14 (Hep II) which binds syndecans (Leiss et al., 2008).

FN fibrils serve as scaffolds facilitating the assembly of other ECM components, influencing its mechanical architecture and regulating signaling to resident cells. This is accomplished through the mosaic of binding sites provided by FN fibrils to a multitude of molecules that in the oncogenic transformation can play critical roles in processes of tumor proliferation, neoangiogenesis, and metastatic invasion. This review examines into the FN regions and principal interacting molecules, exploring their contribution to the structure and biophysical properties of oncogenic microenvironments.

## Cell receptors binding FN play a pivotal role as primary mechanosensors

**FIGURE 2:**
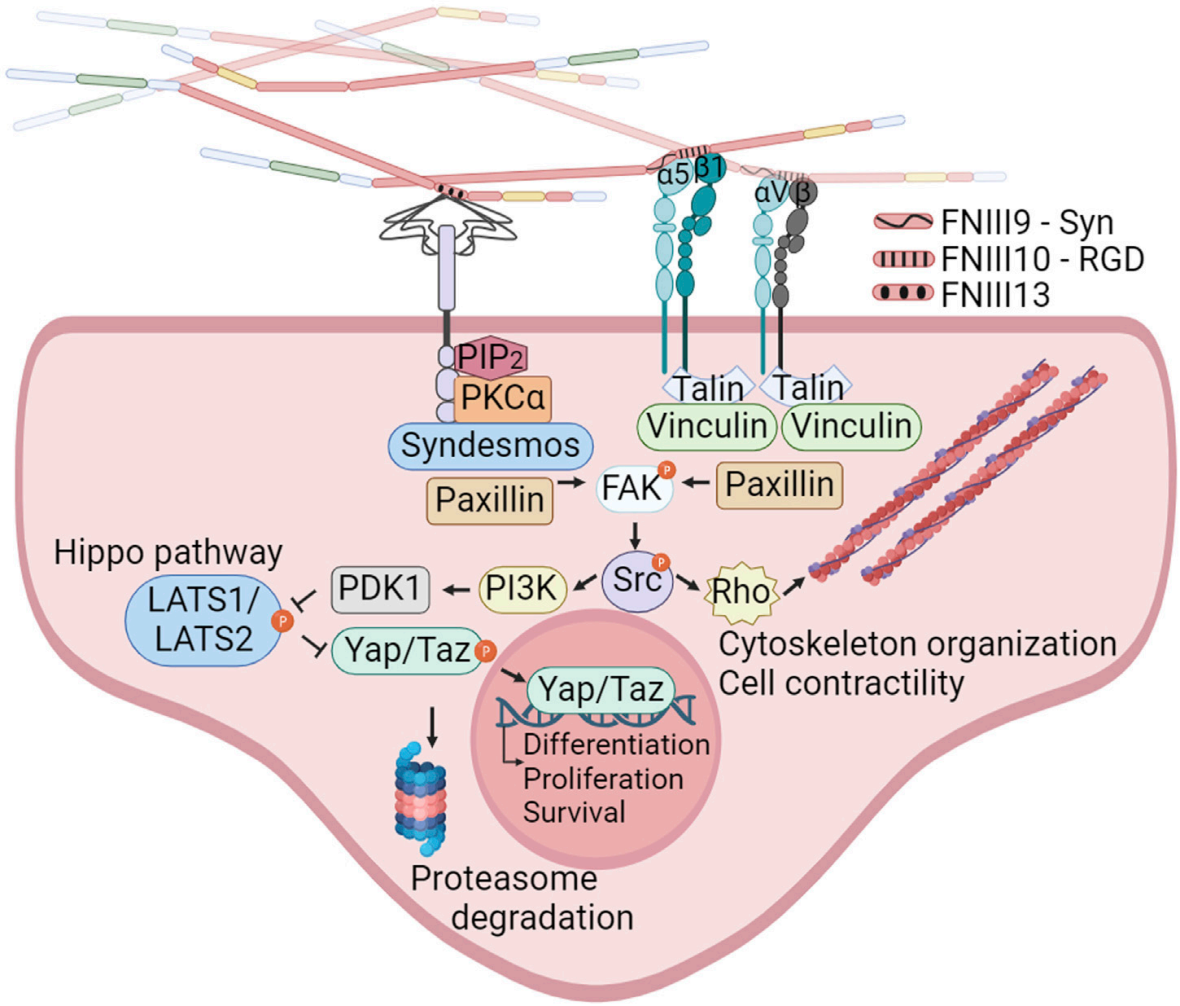
Integrins and syndecans binding FN cooperate in focal adhesion formation, cytoskeleton organization and mechanotransduction. α5β1, αIIbβ3, α8β1 and all the αv-class integrins bind an Arginine-Glycine-Aspartate (RGD) motif in FNIII10. In addition to the RGD motif, α5β1 integrins bind the synergy site in FNIII9 allowing the formation of catch bonds and triggering the engagement of additional integrins. Syndecans bind the heparin II FNIII13-14 region (HepII) by ionic contacts between positively charged amino acids in FN and the negatively charged groups of the heparan sulphate substitutions of syndecans. Integrins binding to the FNIII9-10 repeats, and syndecans binding to the Heparin II modules can stablish cooperative signaling amplifying their mechanoresponses to external forces, including reorganization of the actin cytoskeleton and YAP/TAZ entry into the nucleus. Created with BioRender.com.

Cell binding to FN is mediated by integrins and syndecans (Figure 2). This adhesion triggers both biochemical and bidirectional mechanical signaling between the ECM and the cytoskeleton. About 11 different integrins can bind to FN (Leiss et al., 2008). Among these, at least 8 (α5β1, αIIbβ3, α8β1 and all the αv-class integrins) bind an Arginine-Glycine-Aspartate (RGD) motif in FNIII10. In addition to the RGD motif, FNIII9 harbors the “synergy site”. Unlike the RGD motif, the synergy site is not cell adhesive by itself and has been shown that binds to the α subunit of α5β1 and αIIbβ3 integrins (Bowditch et al., 1994) increasing the lifetime of the integrin bond under mechanical forces, allowing the formation of catch bonds (Friedland et al., 2009; Benito-Jardón et al., 2017). Importantly, the binding of the synergy site by α5β1 triggers the engagement of additional integrins, included αv-class integrins that can withstand higher forces (Strohmeyer et al., 2017). Other regions of FN such as ED-A, two sequences in IIICS, FNIII14 and the FNIII13-14 junction are bound by α4 integrins. The ED-A module also binds α9β1, (Sharma, 1999; Pankov and Yamada, 2002; Leiss et al., 2008). These regions could have an important contribution mediating infiltrated leukocyte adhesion (Guan and Hynes, 1990).

The syndecan family, comprising four members (syndecan 1–4), binds the heparin II FNIII12-14 region (HepII) by ionic contacts between two clusters of positively charged amino acids in FN (in FNIII13 and FNIII14) and the negatively charged groups of the heparan sulphate (HS) substitutions of syndecans (Busby et al., 1995; Sharma, 1999). Syndecans adhesion to FN triggers signaling events, which promote focal adhesions (FAs) assembly and cytoskeleton rearrangement reinforcing integrin signaling and FN fibrils assembly (Woods and Couchman, 2000; Kim et al., 2001; Bass et al., 2007; Mahalingam et al., 2007). The integrin α5β1 cross-talks with syndecans through a cytosolic molecular bridge, between paxillin and syndesmos, generating cooperative signaling between these receptors (Kusano et al., 2000; Denhez et al., 2002; Bass et al., 2007; Chronopoulos et al., 2020; Ahn et al., 2023). Interestingly, the levels of syndecans in cancer cells correlate with tumor size, invasiveness, and metastatic capacity (Kim et al., 2015; Poças et al., 2023). Many of these actions are considered a consequence of the cooperation with integrins (Beauvais and Rapraeger, 2004; Choi et al., 2013).

Cancerous tissues often become unusually stiff as a result of fibrotic changes in the ECM. This stiffness, combined with increased interstitial pressure caused by rapid cell growth and blood vessel leakage, creates an environment that stimulates tumor growth (Basson et al., 2015). This stiffness is implicated in fostering cancer progression through various mechanisms, including the enlargement of FAs (Rubashkin et al., 2014) and modulation of cell contractility. Integrins emerge as critical stiffness-sensors activated by extracellular mechanical forces (Kechagia et al., 2019) and there is ample literature documenting that the increased expression and binding to FN by various integrins, including α5β1, αvβ1, αvβ6, αvβ3, and α9β1, is linked to tumor cell invasion and drug resistance (Koivisto et al., 2000; Barkan and Chambers, 2011; Goodman and Picard, 2012; Gupta et al., 2013; Cooper and Giancotti, 2019; Jang and Beningo, 2019; Li C. et al., 2023; Wu et al., 2023).

The α5β1 integrin, which exclusively associates with FN, enables cells to sense ECM rigidity, translating this mechanical information into the focal adhesion kinase (FAK) activation and subsequent signaling activation of Src kinase, which controls cytoskeletal dynamics and YAP/TAZ translocation to the nucleus. FN-mediated FAK activation is dependent on the mechanical tension transmitted by α5β1 with the contribution of the FN synergy site. In sharp contrast, the ligation between the constitutively exposed binding motif of type I collagen and its receptor integrin α2β1 is tension-independently inducing FAK activation (Seong et al., 2013). Moreover, the interplay between α5β1 and αv-class integrins, upon FN-RGD binding, is essential for cell adaptation to FN fibrils tension (Danen et al., 2002; Schiller and Fässler, 2013; Zaidel-Bar, 2013), with α5β1 stimulating myosin II contractility, while αv-class integrins are immobilized in large FAs providing structural support to cell adhesion (Schiller and Fässler, 2013). This interaction is considered pivotal in the formation of blood vessels in tumors (Kim et al., 2000).

## The FN fibrillar scaffold nucleates ECMs

The FN monomers contain six regions for FN-FN intermolecular interaction (Schwarzbauer, 1991). The secreted soluble FN dimer has a compact conformation mediated by intramolecular interactions. Cytoskeletal forces, generated by actin-myosin contraction and transmitted through integrins, stretch and unfold the FN, generating extended thin fibrils that expose cryptic FN assembly sites (Singh et al., 2010; Schwarzbauer and DeSimone, 2011; Erickson, 2017). FN flexibility allows rotation of individual repeats (Leahy et al., 1996) forming branched networks. Syndecans have been proposed to make the initial contacts with FN by their long HS chains (Woods and Couchman, 1994; Bloom et al., 1999; Klass et al., 2000; Galante and Schwarzbauer, 2007) and subsequently cooperate with integrins bound to FN inducing cytoskeleton contraction and allowing FN fibrils assembly (Huveneers et al., 2008). α5β1 integrins are considered crucial for FN fibril formation. However, αvβ3 integrins can assemble FN fibrils in the absence of α5β1 (Takahashi et al., 2007; Girós et al., 2011). FN lacking a functional RGD site can partially be unfold and assembled into fibrils by syndecans (Bultmann et al., 1998; Benito-Jardón et al., 2020), although the fibrils were dysfunctional (Benito-Jardón et al., 2020). The distribution of traction forces generated by the combination of different receptors will be, therefore, important for determining the final network structure and thus, the molecules that will incorporate to develop a mature matrix (Lemmon et al., 2009). In addition, FN fibrils will be enzymatically crosslinked and grow in length and thickness acquiring variable rigidity.

**FIGURE 3:**
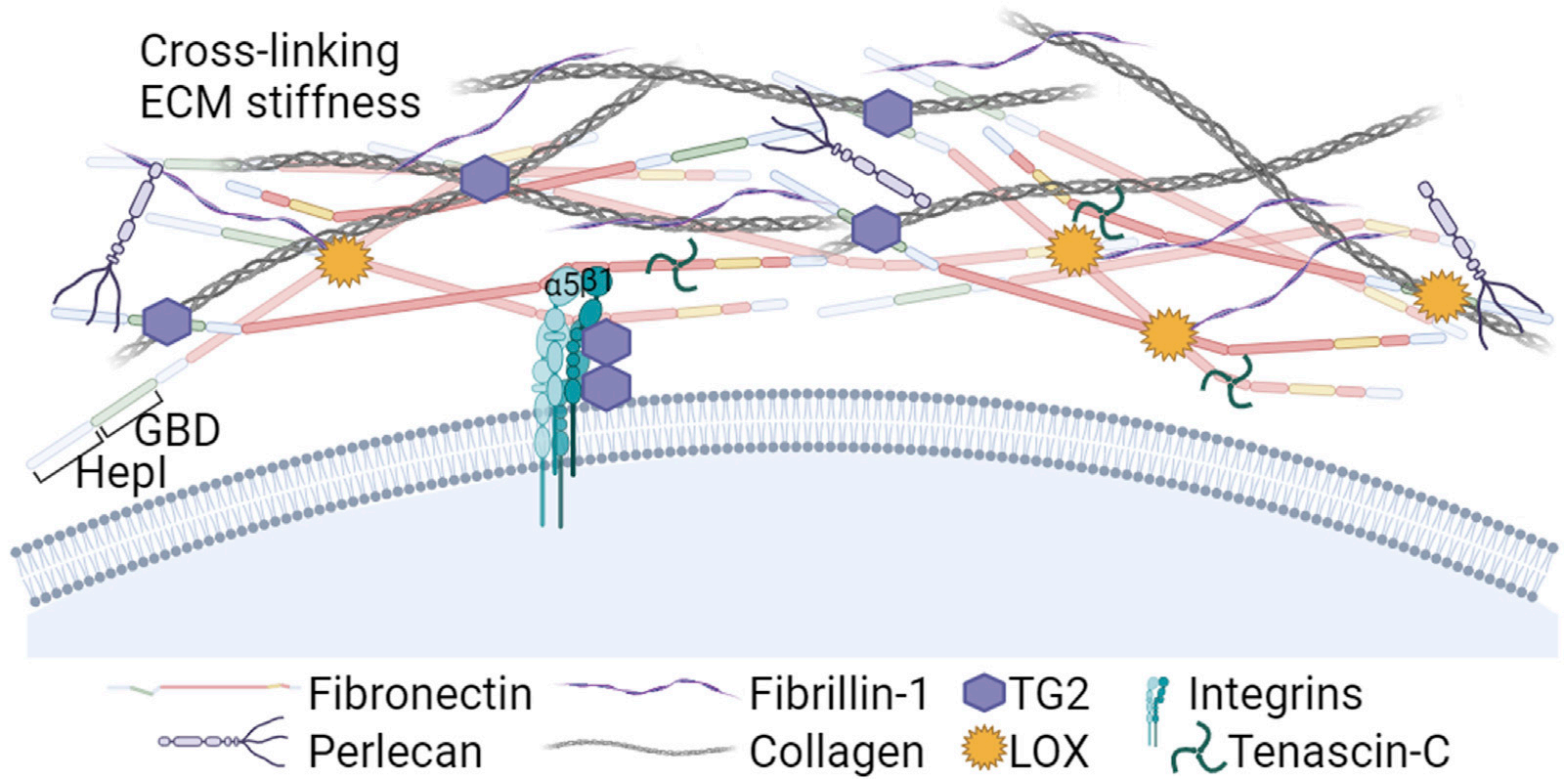
The Heparin I and Gelatin/Collagen binding regions in the N-terminal part of FN: Roles in ECM assembly and crosslinking. The Heparin I region nucleates soluble proteoglycans and fibrin, and the Gelatin Binding Domain (GBD) plays a crucial role interacting with collagens type I and III. Additionally, the N-terminal part of FN binds Lysyl oxidases (LOX) and transglutaminase 2 (TG2). LOX enzymes activity induces FN clustering, leading to increased RGD density and FN fibril formation. In addition, LOX contributes to the crosslinking of collagen, a critical step for ensuring structural stiffness to the ECM. TG2 is a cell co-receptor that forms complexes with the β subunit of integrins. Extracellular TG2 binds FN fibrils and is involved in crosslinking ECM components and LTBPs with FN and fibrillin-1, further stabilizing the ECM and promoting TGFβ activation. Created with BioRender.com.

In tumors, ECM is intensively remodeled and its composition differs from normal tissues and enables new interactions that affect the function of cancer cells such as migration and growth (Figure 3). For example, FN, tenascin-C (TNC), and type I collagen were described to act as pro-metastatic cues (Aguirre-Ghiso et al., 2001; Barkan et al., 2010; Oskarsson et al., 2011), or in patients with breast cancer it was reported that an ECM signature consisting of fibrinogen, elastin, FN, and vitronectin predicts the outcome of the disease (Li S. et al., 2023). Here we focus on the proteins that are more ligated to FN in oncogenic microenvironments.

In many cancers, the tumor stroma is enriched in collagens I and III (Egeblad et al., 2010; Shields et al., 2012). Collagens bind the FN GBD region (McDonald et al., 1982) and its deposition is dependent on the presence of a previously established FN matrix (Sottile et al., 2007; Kadler et al., 2008; Kubow et al., 2015), indicating that FN matrix is an integral part of the collagen fibrillogenesis. In turn, the fibrillar collagen scaffold regulates FN fibrils organization (Dzamba et al., 1993).

During cancer development, the ECM undergoes persistent remodeling characterized by FN and collagen degradation, deposition, and cross-linking. High expression of matrix remodeling genes and cross-linkers is also predictive of a poor prognosis in cancer (Slattery et al., 2013). Among cross-linkers, lysyl oxidases (LOX) play a crucial role in ECM stiffening. Experimental reduction of LOXs prevented MMTV-Neu-induced fibrosis and lowered tumor incidence in a mouse model (Levental et al., 2009), and reduced invasion of glioma cells (Laurentino et al., 2021). In addition to collagen, recently LOX was shown to oxidate FN lysine residues prior to the fibril assembly, inducing FN clustering and leading to increased RGD density and integrin grouping promoting FN fibrillation (Melamed et al., 2023). The aberrantly elevated expression and activity of LOX enzymes that has been reported in several cancer types, predominating in invasive types (Barker et al., 2012), may be a relevant factor accelerating formation of FN-rich heavily cross-linked ECMs around tumors.

Other enzymes bound by FN, such as bone morphogenetic protein 1 (BMP-1) and tissue transglutaminase 2 (TG2) can also stimulate FN-collagen fibril formation. Assembly of the collagen fibrils requires the proteolytic processing of procollagen. Saunders and Schwarzbauer (2019) identified a BMP-1 binding site in the FN HepII domain, whose effect was enhanced by heparin. FN binding of BMP-1 proteinase enhances its processing activity against type I procollagen accelerating its fibril formation (Huang et al., 2009).

TG2 is a multifunctional protein that can be found in the cytosol, in the nucleus, at the cell surface and in the ECM (reviewed by Telci and Griffin, 2006). TG2 forms complexes with β1 and β3 integrins inside the cell during their transport, accumulates on the surface in FAs and functionally collaborates with these receptors, increasing cell contractility (Akimov et al., 2000; Janiak et al., 2006; Chen et al., 2010; Bordeleau et al., 2019) and mediating cell binding to FN via interaction with the FN GBD (Selcuk et al., 2023a). This interaction prevented the anoikis due to the lack of RGD-dependent adhesion (Verderio et al., 2003). In healthy tissues, most secreted TG2 is bound to FN fibrils and catalytically inactive, but will be activated by matrix remodeling (Siegel et al., 2008). Activated TG2 alters the ECM properties by enzymatically cross-linking ECM proteins (Martinez et al., 1994; Stephens et al., 2004; Fortunati et al., 2014) that leads to ECMs stiffening, induces platelet derived growth factor receptor (PDGFR)-integrin association (Zemskov et al., 2009) and cross-links the latent transforming growth factor β binding proteins (LTBPs) to FN and fibrillin, thus promoting the transforming growth factor β (TGFβ) activation (Kumar et al., 2010; Ayinde et al., 2017; Lockhart-Cairns et al., 2022). Increased TG2 expression in several types of cancer has been linked to invasiveness in collaboration with α5β1 integrins (Caffarel et al., 2013) promoting cell adhesion, spreading and contributing to FAs enlargement and FN fibril formation (Akimov et al., 2000; Akimov and Belkin, 2001). TG2 was also linked to cancer cell survival, poor prognosis and chemotherapy resistance (Akimov and Belkin, 2001; Grigoriev et al., 2001; Iacobuzio-Donahue et al., 2003; Martinet et al., 2003; Mangala et al., 2007; Chen et al., 2010; Meshram et al., 2017; Lee et al., 2018; Valdivia et al., 2023).

Dysregulated RNA splicing is a molecular feature that characterizes almost all tumor types and arises from both recurrent mutations and altered expression of trans-acting factors governing splicing (Bradley and Anczuków, 2023). One of the most consistent isoform changes in the ECM of tumors is the upregulation of TNC and of FN splice isoforms (Chiquet-Ehrismann et al., 1991; Orend and Chiquet-Ehrismann, 2006). TNC is a hexameric extracellular matrix glycoprotein. High TNC levels in tumors are linked with increased invasion, metastasis, and often shorter patient survival (Saupe et al., 2013; Gocheva et al., 2017). Inhibiting TNC expression by tumor cells reduces proliferation and can reverse the mesenchymal phenotype to epithelial cells (Wawrzyniak et al., 2020). TNC and FN ED-B are significant components of the angiogenic vasculature in tumors, but are scarce in quiescent adult vessels. TNC is associated with an increase in leaky blood vessels in tumors (Saupe et al., 2013; Rupp et al., 2016; Sun et al., 2019). TNC deposition is also present in the peripheral margins of invasive carcinomas (Giuffrida et al., 2004; Nagaharu et al., 2011). TNC was shown to have an anti-adhesive effect as it induces cell rounding *in vitro*, suppresses actin stress fibers, and promotes actin-rich filopodia formation. These changes were linked to the suppression of RhoA activation and increased endothelin receptor type A (EDNRA) expression (Wenk et al., 2000; Lange et al., 2007). In addition, TNC binds to FNIII13, blocking syndecan-4 binding to FN (Huang et al., 2001), which could contribute to the described changes in matrix patterning and may alter growth factor/chemokine sequestration and presentation (Radwanska et al., 2017). The TNC anti-adhesive effect has shown to affect differently to fibroblasts from tumor cells. In normal cells, TNC slows cell cycle progression (Orend et al., 2003), while in tumor cells, it stimulates proliferation and migration (Huang et al., 2001; Wawrzyniak et al., 2020). The interplay between FN and TNC in tumor angiogenesis is complex and puzzling. While not expressed by endothelial cells, TNC exposure stimulates Wnt signaling and FN expression, promoting the assembly of a dense, branched matrix that supports tubulogenesis, reinforces cell-cell junctions, and protects against anoikis (Radwanska et al., 2017). Additional pro-tumoral actions of TNC include the release of a fragment, activated by MMP-2 processing, that binds the ectodomain of syndecan-4, inducing α5β1 integrin activation, anoikis resistance and cell proliferation and migration (Saito et al., 2007). Moreover, TNC binds LTBPs (Aubert et al., 2021), which in turn releases TGFβ that stimulates epithelial-to-mesenchymal transition (EMT) (Takahashi et al., 2013).

Other ECM components such as perlecan that bind FN are upregulated in tumors and play important roles in the oncogenic microenvironment due to their capacity to bind and cooperate in growth factor (GF) activation (Iozzo and Sanderson, 2011), or Fibrillin1 that binds FN HepI and traps LTBPs, contributing to TGFβ release (Cierna et al., 2016; Ma et al., 2016; Lien et al., 2019; Wang et al., 2022).

## Oncofetal FN, the FN splicing isoforms

Three sites of alternative splicing within the FN molecule have been identified: ED-A, ED-B and IIICS (Goossens et al., 2009). The term oncofetal FN (onfFN) was coined by Matsuura and Hakomori, (1985) to describe a specific FN recognized by the FDC6 monoclonal antibody. This antibody targets an epitope formed by the addition of an O-glycan to the threonine residue in the VTHPGY sequence at the IIICS domain. OnfFN, abundant in fetal and cancer tissues but scarce in normal adult tissues, has expanded its definition and includes the FN isoforms containing ED-A or ED-B (Singh et al., 2010). These isoforms are highly expressed in fetal tissues and solid tumors, highlighting their significance in these contexts.

In studies of human prostate epithelial cell lines, it was observed that TGFβ treatment upregulates onfFN and GalNAc transferase (GALNT6) activity, which is responsible for O-glycosylation of the IIICS domain. This upregulation is linked to enhanced transformational potentials in mammary epithelial cells, promoting cell proliferation (Freire-de-Lima et al., 2011). O-glycosylated onfFN is also expressed by M2-polarized macrophages (da Costa Santos et al., 2023). However, the specific mechanisms by which onfFN contributes to malignancy remain unclear. It is suggested that FNIIICS O-glycosylation could interfere with FN degradation, thus stabilizing the molecule (Park et al., 2011).

**FIGURE 4:**
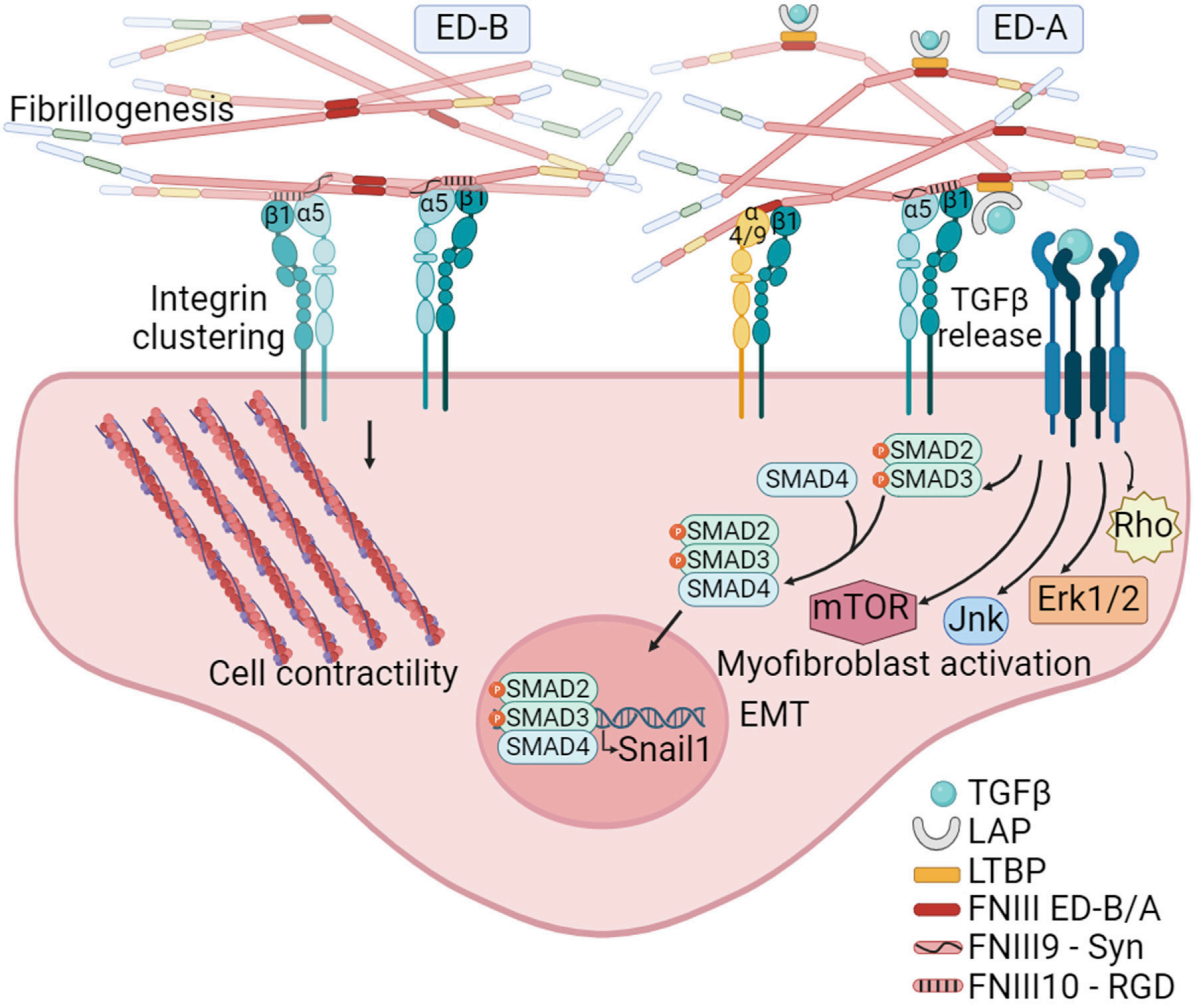
The FN isoforms generated by alternative splicing can impact on FN fibrillogenesis. The inclusion of the ED-B, located between FNIII7 and III8 modules, facilitates the formation of tightly packed head-to-tail homodimers. This structural arrangement promotes simultaneous access to the synergy and RGD motifs on FN, leading to the clustering of integrins, and thus to FN fibril formation and mechanosignaling. The inclusion of ED-A, between FNIII11 and III12 modules, in addition to facilitate fibrillogenesis, also provides specific binding sites to α4β1 and α9β1 integrins expressed by hematopoietic cells. Moreover, ED-A offers a binding site for the LTBP-1. The interaction with LTBP allows for the storage, and TGFβ1 release and signaling under conditions of fibril strengthening or proteolytic degradation. Created with BioRender.com.

The analysis of tumor matrixomas from 435 patients revealed that both ED-A and ED-B FNs are major and essential components of the matrix produced by CAFs in head and neck squamous cell carcinomas (HNSCC). Their presence correlates with poor prognosis (Gopal et al., 2017). The FN ED-B isoform is present at the abluminal sites of endothelial linings in newly formed blood vessels and is prevalent in almost all human solid cancers, lymphomas and some leukemias, and absent in normal tissues. A negative correlation exists between FN ED-B expression and patient survival (Hall et al., 2023). Crystallographic studies have shown that the insertion of ED-B induces a significant twist in the longitudinal orientation of FN monomers, facilitating the formation of tightly packed head-to-tail homodimers (Figure 4). This unique conformation allows simultaneous binding to two integrins with both the RGD and the synergy motif remaining accessible, potentially promoting α5β1 integrin clustering and mechanosignaling (Schiefner et al., 2012).

In contrast to ED-B, ED-A FN is abundant in non-malignant tissues during healing and fibrosis (Serini and Gabbiani, 1999; Kelsh et al., 2015) and its expression together with LTBP-1 is essential for myofibroblast activation (Klingberg et al., 2018). In the context of tumors, ED-A FN plays a crucial role in establishing a metastasis-permissive stromal architecture (Hall et al., 2023), and its expression is closely linked to TGFβ1 activity (Figure 4). A correlation between SNAIL1 levels, a target of TGFβ, and ED-A FN expression has been observed in epithelial cancers. Notably, the absence of the ED-A domain prevents lung metastasis in a mouse breast cancer model (Franco-Valls et al., 2023). FN ED-A is known to bind α4β1 and α9β1 integrins, which are highly expressed on activated neutrophils (Dhanesha et al., 2020). ED-A has been identified as an endogenous damage associated molecular pattern (DAMP) molecule, triggering innate immune responses (Ambesi et al., 2022), suggesting its significant role in tumor infiltration. Further studies have highlighted the potential of FN ED-A in promoting metastasis. A study from Gopal et al. (2017) demonstrated that the migration of head and neck squamous cell carcinoma collectives was facilitated through the engagement of αvβ6 and α9β1 integrins. Beyond ED-A potential role facilitating cancer cell migration and leukocyte infiltration, the inclusion of ED-A may also play a pivotal role in FN fibril formation. This is based on the observation that, under normal physiological conditions, pFN lacks the ED-A domain.

## The FN HepII region binds growth factors

**FIGURE 5:**
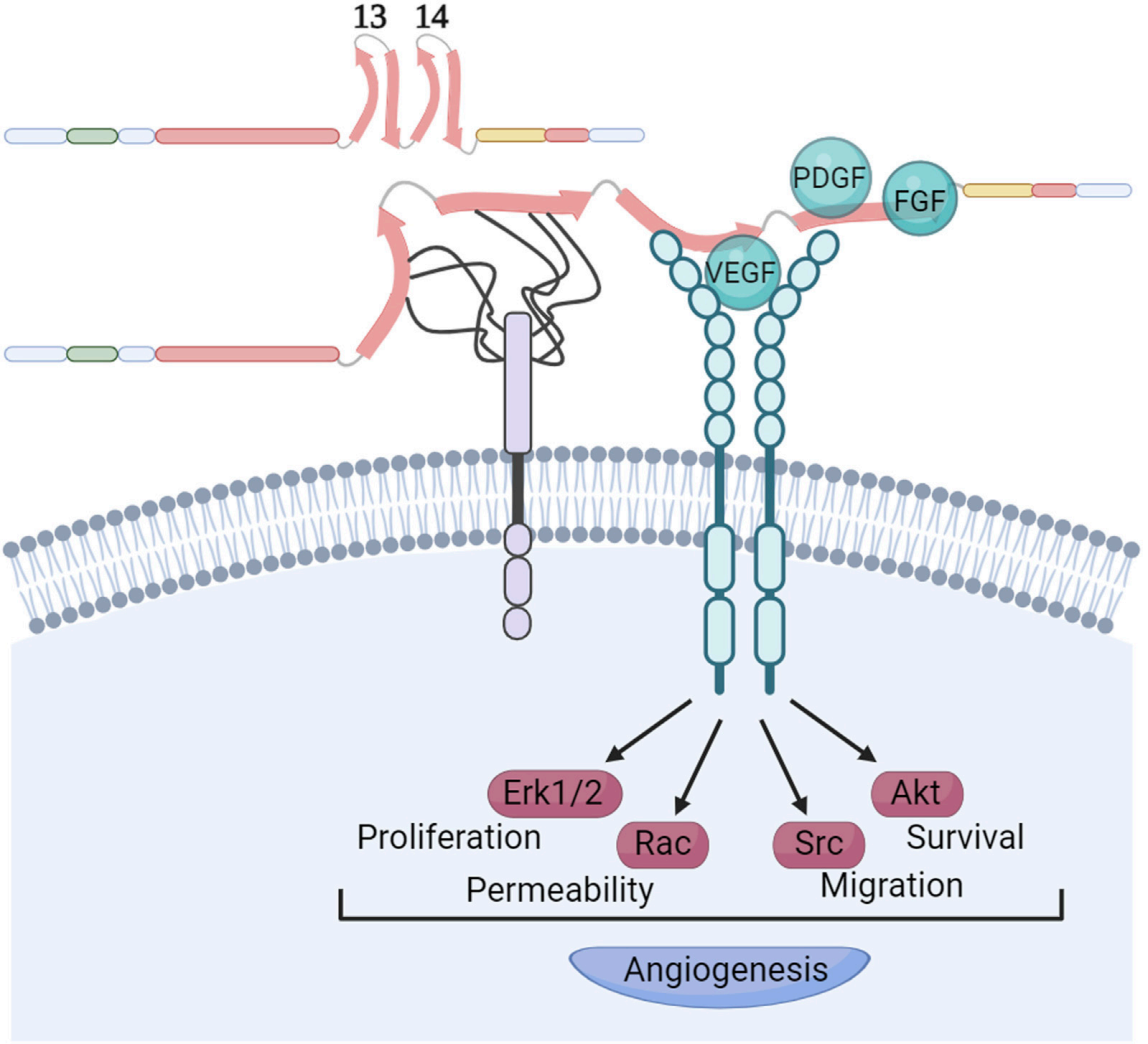
Syndecans modulate growth factors recruitment to FNIII14 region. The HepII region of FN exhibits a remarkable affinity for binding growth factors. This interaction occurs in a manner that apparently is modulated by the presence of syndecans, as well as the elasticity of the ECM. The chemical structure and length of the heparan sulphate chains of syndecans determine the strength of their interaction with the FNIII13 and FNIII14 domains. It is known that these interactions facilitate the opening of the FNIII14 region, enabling the recruitment of VEGF and its receptor. This recruitment is essential for stimulating VEGFR activation, a critical step in promoting angiogenesis. Other growth factors binding the FNIII14 region like PDGF and FGFs might use similar mechanisms for activation of their receptors. Created with BioRender.com.

The interplay between growth factor (GF) signaling and FN-binding integrins is a pivotal regulator of cellular signal transduction within the tumor ECM. The HepII region binds GFs profusely in a fashion apparently modulated by syndecans and by the ECM elasticity (Figure 5). The repertoire of GFs that can bind HepII includes several representatives of the platelet-derived growth factor (PDGF), vascular endothelial growth factor (VEGF), fibroblast growth factor (FGF) and TGFβ superfamilies (Martino and Hubbell, 2010). Wijelath et al. (2006) demonstrated that the physical linkage of the RGD and HepII regions is both necessary and sufficient to promote endothelial cell proliferation, migration, and ERK activation induced by VEGF. It was shown that the VEGF does not directly bind to HS; rather, the HS chains of syndecans facilitate the transformation of FN into an open conformation which in turn allows the GF binding to the FNIII14 region (Mitsi et al., 2006). Furthermore, the efficacy of heparin/HS to enhance VEGF binding to FN is modulated by its chemical composition and chain length. Hypoxia and low extracellular pH, conditions known to stimulate the formation of new blood vessels, trigger alterations in the chemical structure of HS produced by endothelial cells. These changes are closely associated with enhanced accumulation of VEGF in FN. This process, in turn, facilitates the interaction between VEGF and its receptor VEGFR2, effectively promoting angiogenesis (Goerges and Nugent, 2004; Buczek-Thomas et al., 2019).

It is well established that GFs bound to FN retain the ability to bind to their respective receptors. Usuelli et al. (2021) demonstrated that FN provides interaction motifs to the extracellular domain of the VEGF receptor 2 (VEGFR2), akin to the VEGF-binding sites that were also exposed upon heparin-induced conformational changes in FN. This suggests that a triple complex formation could occur, initiated either by VEGF or VEGFR2 binding to FN, followed by the recruitment of the third binding partner, thereby activating angiogenic signaling pathways. While most of these studies focused on VEGF, it remains an open question whether this activation mechanism is common to other GFs that bind the HepII region. Therefore, factors like matrix stiffness and the composition of syndecan HS chains are crucial in inducing conformational changes in FNIII13-14, thereby controlling GF interactions with FN, bioavailability and spatiotemporal cellular signaling.

The FN HepII region also binds several members of the TGFβ superfamily such as TGFβ1, BMP-1, BMP-2 and -7 (Martino and Hubbell, 2010). TGFβ triggers the EMT in epithelial-origin cancers. This transition leads to the development of pro-metastatic traits, such as a fibroblastic morphology, diminished expression of epithelial markers and increased expression of mesenchymal markers including FN and matrix-metalloproteinases (MMPs), which collectively enhances cell motility. In the stroma, TGFβ drives the transformation of CAFs into highly contractile myofibroblasts that express αSMA and secrete substantial quantities of FN and collagen I. Disrupting TGFβ1 is linked to reduced macrophage polarization to M2 and associated with reduced tumor growth (Zhou et al., 2023). TGFβs follow a complex activation mechanism: cells secrete TGFβs noncovalently bound to the latency-associated propeptide (LAP), which in turn attaches to LTBP, stored in the ECM bound to various molecules. Fibrillin-1 binds LTBP-3 and -4, while FN fibrils store LTBP-1 (Zilberberg et al., 2012), which bonds to ED-A and the FN HepII region. Unlike other GFs, LTBP-1 binding to FN HepII can be hindered by HS (Griggs et al., 2017), suggesting that FN ED-A might boost LTBP-1 incorporation into the FN matrix (Klingberg et al., 2018). Inhibiting FN fibrillogenesis in malignant breast cancer cells blocked TGFβ activation and signaling, thus impeding EMT (Griggs et al., 2017). The active TGFβ1 form is released either via MMP-mediated cleavage of the ECM or by cell-induced FN fibrils strengthening leading to conformational changes in LAP (Buscemi et al., 2011; Shi et al., 2011; Klingberg et al., 2014), highlighting the contribution of CAFs to TGFβ release. TG2, which has been shown to cross-link fibrillin and LTBPs and to stimulate cell contractility (Bordeleau et al., 2019), enhances TGFβ activation (Lockhart-Cairns et al., 2022).

Among BMPs, BMP-2 and BMP-7 exhibit robust interactions with the HepII domain of FN (Martino and Hubbell, 2010). BMP-2, commonly overexpressed in diverse cancers and tumor cell lines, predominantly contributes to processes such as in metastasis, EMT and invasion (Singh and Morris, 2010; Wu et al., 2022), whereas BMP-7 acts as an inhibitor of metastasis in certain cancers like melanoma (Na et al., 2009). The involvement of distinct BMPs in either promoting or inhibiting tumor progression hinges on BMP dosage, microenvironment and genetic background of the cell. The binding to FN emerged as a potential regulatory element influencing the action of BMPs. It has been shown that the secretion of FN by cells is critical for BMP-2-mediated signaling (Fourel et al., 2016). Furthermore, several studies have shown that FN-bound BMP-2 regulates cellular behavior in a manner distinct from soluble BMP-2. This effect was attributed to the close proximity and interplay of integrin-binding and BMP-2-binding domains within FN (Crouzier et al., 2011; Wei et al., 2015).

## FN in tumor cell dissemination and dormancy

Distinct tumors exhibit varied mechanisms of dissemination, highlighting the intricate nature of cancer progression. One crucial factor is the FN present in the migration tracks employed by metastatic cells (Erdogan et al., 2017). However, FN role in dissemination extends beyond migration tracks. A compelling illustration is found in high-grade serous ovarian cancer (HGSOC), where metastasis to the abdominal space occurs through the formation of tumor cell aggregates that contain CAFs, and FN expressed by CAFs plays a crucial role in the aggregation process. Importantly, FN expression was dependent on PDGFR-β (Gendrau-Sanclemente et al., 2023). On the other hand, it has been described that TG2 and cross-linked FN within extracellular vesicles (EVs) produced by metastatic breast cancer cells, determines the formation of the metastatic niche in lungs (Shinde et al., 2020). Vascular invasions are described as clusters of proliferating epithelial tumor cells enveloped by a luminal endothelial cell monolayer and by Fsp1-positive fibroblasts containing FN, laminin and TNC between the two stromal cell layers. The endothelial layer integrity would support tumor cell survival and overall metastasis (Sun et al., 2019). This multifaceted involvement of FN in diverse mechanisms of dissemination emphasizes its significance for cancer metastasis.

Cancer cells disseminate from primary tumors and establish themselves in distant organs, where they can lay dormant or quiescent for extended periods before manifesting detectable metastases. The dynamic interplay between ECM-derived mechanical forces and composition significantly influences specific cell states within tumor tissues. These factors, in turn, dictate the likelihood of tumor relapse. Increased matrix stiffness and aligned fibers are identified as hallmarks in various cancers, such as breast, pancreatic or colorectal cancers. Microenvironments mirroring the normal softness of healthy tissues can impede oncogene-mediated cell reprogramming and tumor emergence. However, certain oncogenes, such as RTK-Ras, confer a disproportionate cellular response to even subtle changes in ECM rigidity, converting cells into tumor-initiating cells (Panciera et al., 2020), a process that has been assigned to YAP/TAZ mobilization to the nucleus induced by mechanical signals.

Dormancy and the transition from dormancy to proliferative metastatic growth involve complex mechanisms. Three-dimensional cell culture environments are shown to induce quiescence (Barkan et al., 2008), and cancer cells needed to organize a fibrillar FN matrix to maintain quiescence status (Barney et al., 2020). However, stiff surfaces promote cell proliferation. Interestingly, following treatment with cisplatin, surviving hepatocellular carcinoma cells from soft substrates had significantly higher clonogenic capacity than surviving cells from a stiff microenvironment (Schrader et al., 2011; Kondapaneni and Rao, 2020). In breast, prostate, melanoma, and fibrosarcoma cell lines, the ERK/p38 activity ratio was used to predict the *in vivo* behavior, with a high ratio favoring tumor growth and a low ratio inducing tumor dormancy. The ERK/p38 ratio was under the regulation of the urokinase plasminogen activator receptor (uPAR) complexed with α5β1 integrins leading to integrin activation, which facilitates the formation of FN fibrils and activates ERK (Aguirre-Ghiso et al., 2001; Aguirre-Ghiso et al., 2003). ECM degradation driven by MMP-2 (Barney et al., 2020) or MMP-9 (Albrengues et al., 2018) also was shown to disrupt dormancy. ECM proteomics of human head and neck squamous cell carcinomas and murine mammary tumors identified collagen III and I as key contributors to dormancy induction and maintenance *in vivo* (Qiu et al., 2020; Di Martino et al., 2022). Interestingly, non-canonical Discoidin Domain Receptor Tyrosine Kinase (DDR1) signaling mediated by collagen I/III was implicated in cancer stem cell self-renewal and metastatic reactivation (Gao et al., 2016; Di Martino et al., 2022). Definitively, abundant evidences point that matrix remodeling activities contribute to interrupting dormancy.

## Conclusion

FN plays an essential role in the assembly of ECMs, and FN fibrils orchestrate signals that govern specific cell states within tumor tissue, included metastatic reawakening. Their multifaceted interactions with various ECM components and cell receptors, as well as their capacity to undergo mechanical stretching by cell receptors affects ECM rigidity and growth factor storage and activation.

However, the role of FN in tumor genesis and malignant dissemination is still under debate (Beier et al., 2007). In some cancers, FN expression can act as a tumor suppressor, as observed in tyrosine kinase receptor Met and its ligand, hepatocyte growth factor (HGF)/scatter factor-mediated tumorigenesis (Taylor et al., 1998). *In vitro* studies have shown that tumor cells surrounded by FN fibrils, which support CAFs, experience reduced proliferation and YAP nuclear export due to the compressive forces exerted by CAFs (Barbazan et al., 2023). In breast cancer cells, autocrine FN expression by tumor cells that have undergone EMT is associated with a non-metastatic phenotype, yet the FN produced by them contributes to the invasion and metastasis of their epithelial counterparts. Moreover, genetic depletion of FN expression allows tumor cells to regain epithelial characteristics and initiate lung tumor formation, highlighting the concept of epithelial-mesenchymal heterogeneity in promoting cancer metastasis (Shinde et al., 2018).

The tumor ECM is typically fibrotic and stiff. An unresolved question is, however, whether FN fibrils in the tumor ECM are strengthened. Recent studies using a tension nanoprobe specific to the relaxed FN GBD region showed that FN fibers are under high tension in healthy mouse organs, whereas tumor tissues have a higher content of relaxed fibers (Fonta et al., 2020). Interestingly, collagen I (Kubow et al., 2015) and TG2 (Selcuk et al., 2023b) can only bind to relaxed FN GBD, and binding is lost when FN is strengthened. Moreover, once assembled, collagen fibrils prevent FN fibrils from being stretched by cellular traction forces (Kubow et al., 2015). These results would suggest that relaxed FN fibrils in the tumor might promote collagen and TG2 assembly, and other factors such as collagen abundance itself and cross-linkers would contribute to tumoral ECM stiffness (Egeblad et al., 2010). The abundance of relaxed FN GBD in tumors could result from intense proteolytically cleaved FN fibers and, otherwise, does not preclude that the rest of the molecule remains relaxed. Unlike type I and II modules, FN type III repeats lack intradomain disulfide bonds. This structural difference endows them with an elastic conformation capable of absorbing significant tension before strengthening the N-terminal part of the FN dimers. Integrins are organized in nanoclusters within FAs, with an optimal spatial arrangement for effective mechanotransduction (Jain et al., 2023). Then, the integrin-binding parts of FN are located in the middle of the FNIII region and could transmit minimal variations on the tension supported by the fibrils. In this line, it is unknown whether the double interaction of the α5β1 integrin with the RGD motif and the synergy site is favored with the conformation that FNIII9-10 acquires in tumors. The inclusion of ED-B, but also ED-A, TG2 adhesive contribution, or collagen fibers conformation might promote this interaction.

The most consistent cancer-specific feature is the exceptional or overexpression of oncofetal FNs, as opposed to their expression levels in normal tissue. Another question is whether the expression of oncofetal FNs and TNC results from oncogenic mutations or is induced by components like TGFβ in the tumor microenvironment, as observed with oncfFN in human prostate epithelial cell lines (Freire-de-Lima et al., 2011).

Currently, many efforts are ongoing to develop small molecule inhibitors targeting TG2-FN interface (Yakubov et al., 2014; Sima et al., 2019), block FN extra domains particularly ED-B (Menrad and Menssen, 2005; El-Emir et al., 2007; Sauer et al., 2009; Schliemann et al., 2009; Zhang et al., 2022) or impair FN exocytosis to reduce tumor migration and invasion (Park et al., 2022). Among the integrin blockers, Cilengitide is a cyclic pentapeptide containing RGD that blocks αvβ3 and αvβ5 integrins, has been studied as anti-angiogenic in diverse tumors in mouse and reached to phase III, but stopped, in patients with glioblastoma (Desgrosellier and Cheresh, 2010). Other α5β1 integrin inhibitors include velociximab, an α5β1 function-blocking murine antibody (Ramakrishnan et al., 2006) and ATN-161 (Livant et al., 2000), which was initially designed to block the synergy interaction, although later was shown to bind to the β subunits of several integrin heterodimers, including α5β1, αvβ3, and αvβ5 (Donate et al., 2008). We demonstrated that fibroblasts lacking FN expression exhibited a decrease in phosphorylated FAK (pY397-FAK) when cultured on FN with an impaired synergy sequence (FNsyn), compared to cells on wild-type FN (Benito-Jardón et al., 2017). Furthermore, we also showed that the inactivation of the synergy site was partially compensated by αvβ3 integrins. Additionally, skin wounds in mice genetically modified to express FNsyn (*Fn1* ^*syn/syn*^ mice) displayed reduced TGFβ1-mediated cell signaling (Gimeno-LLuch et al., 2022). Altogether these data suggest the potential of targeting the synergy region, either alone or in conjunction with inhibitors of αv-class integrins, as a strategic approach to disrupt α5β1 integrin-mediated mechanotransduction in tumors.

Finally, an interesting area for future research is investigating the potential of FN to attract leukocytes that express α4β1/β7 and α9β1 integrins, particularly in the context of cancer progression. Moreover, to potentiate this effect in conjunction with check-point immunotherapy, especially when targeted using peptides containing RGD, RGE or FN synergy sequences, may prove to be a highly effective strategy to combat cancer progression.

## Figures and Tables

**Table:** 

**ECM**	extracellular matrix
**CAFs**	cancer-associated fibroblasts
**FN**	fibronectin
**αSMA**	α-smooth muscle actin
**pFN**	plasma FN
**cFN**	cellular FN
**GBD**	gelatin binding domain
**HepI**	heparin I binding site
**ED-A**	extra domain A
**ED-B**	extra domain B
**HepII**	heparin II binding region
**HS**	heparan sulphate
**FAs**	focal adhesions
**FAK**	focal adhesion kinase
**TNC**	tenascin-C
**LOX**	lysyl oxidases
**BMP-1**	bone morphogenetic protein 1
**TG2**	tissue transglutaminase 2
**PDGFR**	platelet-derived growth factor receptor
**LTBPs**	latent transforming growth factor β binding proteins
**TGFβ**	transforming growth factor β
**EDNRA**	endothelin receptor type A
**EMT**	epithelial-to-mesenchymal transition
**onfFN**	oncofetal FN
**GALNT6**	GalNAc transferase
**HNSCC**	head and neck squamous cell carcinomas
**DAMP**	damage associated molecular pattern
**GF**	growth factor
**PDGF**	platelet-derived growth factor
**VEGF**	vascular endothelial growth factor
**FGF**	fibroblast growth factor
**VEGFR2**	VEGF receptor 2
**MMPs**	matrix-metalloproteinases
**LAP**	latency-associated propeptide
**HGSOC**	high-grade serous ovarian cancer
**EVs**	extracellular vesicles
**uPAR**	urokinase plasminogen activator receptor
**DDR1**	discoidin domain receptor Tyrosine Kinase
**HGF**	hepatocyte growth factor
